# The highly differentiated gut of *Pachnoda marginata* hosts sequential microbiomes: microbial ecology and potential applications

**DOI:** 10.1038/s41522-024-00531-7

**Published:** 2024-07-31

**Authors:** Àngela Vidal-Verdú, Daniel Torrent, Alba Iglesias, Adriel Latorre-Pérez, Christian Abendroth, Paola Corbín-Agustí, Juli Peretó, Manuel Porcar

**Affiliations:** 1 Institute for Integrative Systems Biology I2SysBio (University of Valencia – CSIC). C/ Catedrático Agustín Escardino Benlloch 9, 46980 Paterna, Spain; 2 Darwin Bioprospecting Excellence S.L. C/ Catedrático Agustín Escardino Benlloch 9, 46980 Paterna, Spain; 3 Chair of Circular Economy, Brandenburg University of Technology Cottbus-Senftenberg, Siemens-Halske-Ring 8, 03046 Cottbus, Germany; 4 Department of Biochemistry and Molecular Biology, University of Valencia, C/ Dr. Moliner 50, 46100 Burjassot, Spain

**Keywords:** Applied microbiology, Metagenomics

## Abstract

Insect gut microbiomes play a crucial role in the insect development and are shaped, among other factors, by the specialized insect diet habits as well as the morphological structure of the gut. Rose chafers (*Pachnoda* spp.; Coleoptera: Scarabaeidae) have a highly differentiated gut characterized by a pronounced hindgut dilation which resembles a miniaturized rumen. Specifically, the species *Pachnoda marginata* has not been previously studied in detail in terms of microbial ecology. Here, we show a fine scale study of the highly compartmentalized gut of *P. marginata* by using amplicon and metagenomic sequencing to shed light on the bacterial, archaeal and fungal communities thriving in each section of the gut. We found a microbial gradient along the gut from aerobic (foregut) to strictly anaerobic communities (hindgut). In addition, we have characterized interesting biological activities and metabolic pathways of gut microbial communities related to cellulose degradation, methane production and sulfate reduction. Taken together, our results reveal the highly diverse microbial community and the potential of *P. marginata* gut as a source of industrially relevant microbial diversity.

## Introduction

Animal guts harbor microbial communities that play a pivotal role in the ecology and fitness of their hosts. The gastrointestinal tract is recognized as a vibrant ecosystem teeming with an immense array of microorganisms. These microbial communities, collectively known as the gut microbiota, comprise bacteria, archaea, protists, fungi, and viruses, which have coevolved with their animal hosts. From mammals to birds, reptiles, or insects, the gut microbiota exerts a profound influence on host physiology, immune system development, metabolism, and even behavior^1^. Understanding the composition and functions of this microbial diversity is essential for shedding light on the intricate interplay between animals and their microbiological partners and unraveling the mechanisms underlying host-microbe interactions.

Insects comprise the most diverse group of animals on Earth and harbor an important microbial diversity within their guts^2^. Among the insect orders, Lepidoptera members have been extensively studied for their gut microbiota. These insects exhibit highly specialized feeding habits, where their gut microbiota plays a crucial role in assisting digestion, detoxification, and nutrient acquisition from the feeding material^3,4^. This specialization can result in a low gut microbial diversity like in the case of *Galleria mellonella* which is dominated by *Enterococcus* species^5^. Similarly, Diptera members showcase a remarkable diversity of gut-associated microbes, which have been shown to influence aspects of their physiology, such as immunity and reproductive success^6–8^. Moving beyond, Coleoptera, the largest order of insects, harbor diverse gut microbiomes that have been linked to their ability to exploit a wide range of food sources, including leaves, decaying matter, and even other insects^9^. Therefore, the study of the intricate associations between different insect orders and their gut microbiota may provide insights into ecological processes, evolutionary adaptations, and strategies for their biotechnological exploitation. For example, the gut microbiota of the red palm weevil *Rhynchophorus ferrugineus* (Coleoptera: Curculionidae), a major palm pest, mainly consists of facultative and obligate anaerobic bacteria responsible for palm tissue fermentation^10^. Previous research has also shown that soil microorganisms populate the gut of *Popillia japonica* (Coleoptera: Scarabaeidae) larvae, although as the insect develops, the richness and diversity decrease in correlation with the micro-environments of the different gut sections^11^. Another study, that described and compared the microbial gut communities associated with five xylophagous beetles of the Cerambycidae family concluded that the bacterial and fungal communities varied by beetle species and between individual organisms but were in all cases enriched in microorganisms involved in lignocellulose degradation and nitrogen fixation^12^.

Larvae of rose chafers (*Pachnoda spp.;* Coleoptera: Scarabaeidae) have been studied for their ability to transform lignocellulosic substrates into various fermentation products, some of them used as carbon and energy sources by the host. The gut of the larvae of *Pachnoda* resembles a small bioreactor or a miniaturized rumen, as different specialized microbial taxa are involved in the anaerobic digestion of plant biomass into methane-rich biogas^13^. The intestinal tract of *Pachnoda* species is divided into a short foregut, an elongated midgut and a massive, dilated hindgut. In a bit more detail, the foregut consists of the mandibles and the esophagus; the midgut is a highly alkaline environment with pH ranging from 10–12 that initiates the softening of the lignocellulosic biomass, whereas the hindgut, with a neutral pH, is a highly anoxic niche that harbors the largest part of the microorganisms involved in the fermentation of cellulosic and hemicellulosic materials^14^.

For decades, researchers have investigated the composition and role of the gut microbiota in insects. Already before high throughput DNA sequencing was available, results from culturing techniques suggested substantial differences in the distribution of microbial taxa throughout the various sections of the intestinal tract of insects^15^. In the case of *Pachnoda marginata*, previous works have provided vague or partial descriptions of the composition of its gut microbiota mainly from enriched cultures on wheat straw^14,16^ and sulfate-containing media^17^.

In this work, we describe in depth the bacterial, fungal and archaeal composition of the highly differentiated *P. marginata* gut and we demonstrate the biotechnological potential of the respective microbial contents to degrade cellulose, produce biogas and reduce sulfates.

## Results

**Fig. 1:**
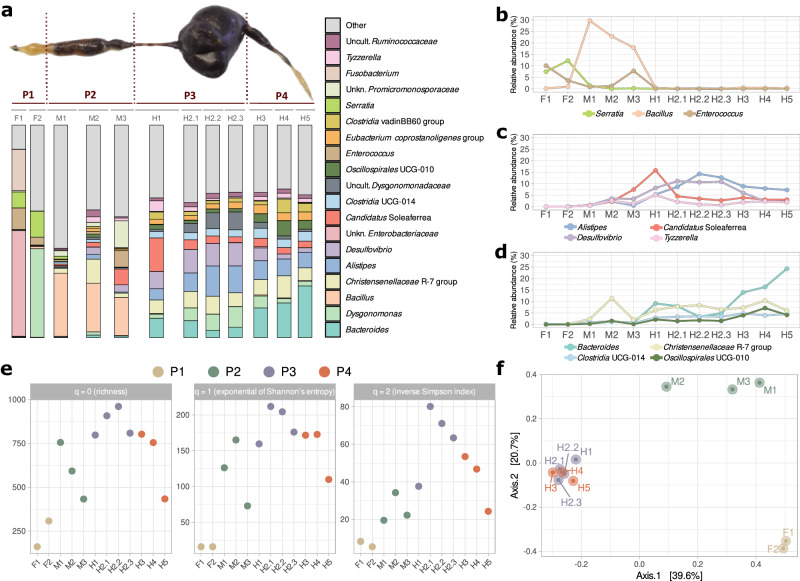
Bacterial community along the gut of *Pachnoda marginata* larvae divided into 12 sections*.* The bacterial portion of the microbiota was analyzed by 16S rRNA gene sequencing. **a** Complete gut dissection of *P. marginata* larvae. The gut in the figure was 6.8 cm long when extended as shown. The gut is divided into 12 sections corresponding to foregut (F1, F2), midgut (M1-M3) and hindgut (H1-H5). Each bar shows the 20 most abundant bacterial genera in each section. **b** Relative abundance of three bacterial genera that were only present along the foregut and midgut. **c** Relative abundance of four bacterial genera that increased in abundance in the first half of the hindgut. **d** Relative abundance of four bacterial genera that increased in abundance in the second half of the hindgut. **e** Alpha diversity metrics, including Hill numbers *q* = 0 (species richness), *q* = 1 (exponential of Shannon’s entropy), and *q* = 2 (inverse Simpson index), based on ASVs with 99.9% similarity. **f** Principal Coordinate Analysis (PCoA) plot based on Bray-Curtis dissimilarities (ASV level); the axes represent the two dimensions that capture the most variation in the community data.

The taxonomic composition of the gut of *P. marginata* was firstly studied using 16S rRNA gene sequencing (regions V3 and V4) of 12 different sections: F1 and F2 (Foregut - P1); M1-M3 (Midgut - P2); H1-H2.3 (first half of the Hindgut—P3) and H3-H5 (second half of the Hindgut—P4) (Fig. 1a). Considerable taxonomic differences were found depending on the region analyzed, with a greater taxonomic homogeneity observed among the sections in the midgut and hindgut (Fig. 1a). In the foregut (F1 and F2 sections) there were few shared taxa among the two sections, such as *Serratia* and *Enterococcus*. However, an unknown genus of the family *Enterobacteriaceae* predominated in F1 and was virtually absent in the rest of the gut, whereas *Dysgonomonas* was the predominant genus in F2. In the midgut sections M1, M2, and M3, *Bacillus* and, to a lesser extent, an unknown member of the family *Promicromonosporaceae* and a candidate genus of the family *Soleaferrea* were prominent. Finally, within the hindgut (H1-H5), the taxonomic composition was balanced, with no major differences among these sections. Furthermore, some of the most abundant genera showed clear trends along the gut. This was the case for *Alistipes, Desulfovibrio*, *Candidatus* Soleaferrea, and *Tyzzerella*, more abundant in the first sections of the hindgut (Fig. 1c); and *Bacteroides*, *Christensenellaceae* R-7 group*, Clostridia* UCG-014 and *Oscillospirales* UCG-010, which were more abundant in the second half of the hindgut (Fig. 1d). They went from being virtually undetectable at the foregut to representing about 50% of the bacterial species detected in the hindgut. Indeed, differential abundance analysis confirmed the significant increase in the presence of all these genera (Supplementary Dataset 1). In contrast, other genera such as *Bacillus*, *Enterococcus* and *Serratia* showed a significant opposite trend, being present in the foregut and midgut, yet completely absent in the hindgut (Fig. 1b).

Alpha diversity analysis showed that the richness of amplicon sequence variants (ASVs) and Hill numbers for *q* = 1 and *q* = 2 were lower in the foregut (Fig. 1e). An average of 234.5 different ASVs (minimum = 161; maximum = 308) were found there, whereas in the other gut sections, between 400 and 1000 ASVs were identified, indicating a higher taxonomic diversity. In terms of beta diversity analysis, the PCoA showed a clear clustering and therefore taxonomic proximity between sections belonging to the same gut segment. The PERMANOVA test confirmed that there were significant differences between the taxonomic compositions of the different gut sections (*p*-value = 1e−3), highlighting the distinct microbial profiles present in each group segment that coincide with the biological division of foregut, midgut, and hindgut (Fig. 1f).

**Fig. 2:**
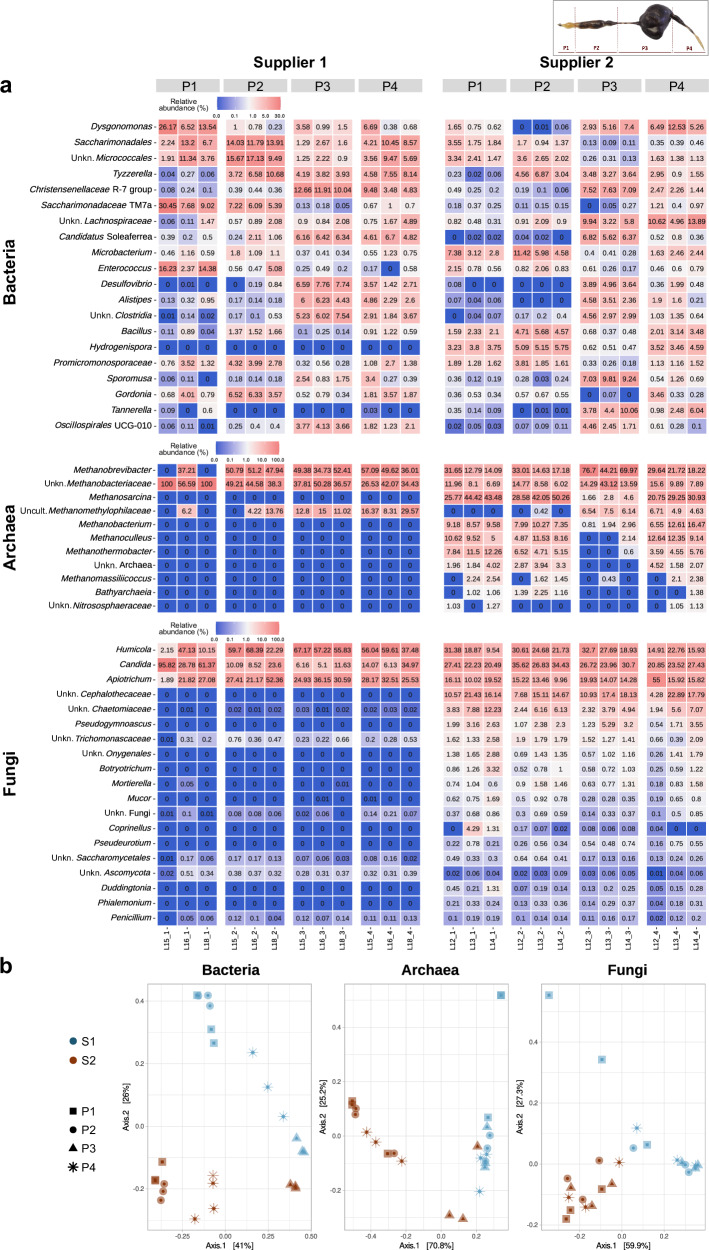
Comparison of the results obtained in the 16S rRNA gene and ITS2 region sequencing analysis according to the supplier. **a** Bacterial, fungal and archaeal communities in each gut section. Three larvae were analyzed per supplier (larvae labeled as number 15, 16 and 18 from S1 and larvae 12, 13 and 14 from S2). **b** Principal Coordinate Analysis (PCoA) plot based on Bray-Curtis dissimilarities (genus level) of bacteria, archaea and fungi in sections P1 to P4 of the gut of *P. marginata* from both suppliers; the axes represent the two dimensions that capture the most variation in the community data.

In parallel, in order to analyze the gut microbiota variability of two different insect populations, the gut microbiota (including bacteria, archaea and fungi) from larvae supplied by two different providers of *P. marginata* was compared by analyzing: Foregut (P1), Midgut (P2), first half of the Hindgut (P3), and last half of the Hindgut (P4) sections. In general, the analyses from both suppliers gave comparable results, so that the bacterial, archaeal and fungal communities that were predominant in the larvae from one supplier were also predominant in the other (Fig. 2a). However, the microbial diversity in terms of number of different ASVs present in larvae from Supplier 2 was considerably higher, in bacteria (532.7 ± 62.1 ASVs in S1 and 859.0 ± 46.7 in S2; *p*-value = 2e−3 in Wilcoxon test), archaea (5.6 ± 0.9 ASVs in S1 and 9.25 ± 0.5 in S2; *p*-value = 3e−3), and fungi (69.7 ± 3.5 ASVs in S1 and 138.8 ± 8.5 in S2; *p*-value = 4e−5) as well as the number of exclusive taxa only present in Supplier 2 (Supplementary Fig. 1). For example, many archaeal genera that were detected in the samples from this supplier were barely detected in Supplier 1, such as *Methanosarcina*, *Methanobacterium* and *Methanoculleus* as well as unknown genera of the *Cephalothecaceae, Chaetomiaceae* families and *Pseudogymnoascus* in the case of fungi. Differential abundance analyses showed that there was a larger number of taxa with statistically different abundances between the two suppliers in the foregut (P1) and midgut (P2), while the comparison of the hindgut sections (P3 and P4) showed smaller significant differences (Supplementary Dataset 2). Beta-diversity analyses reflected these differences in the taxonomic profiles obtained, with samples from the same supplier grouping together (Fig. 2b). The PERMANOVA test confirmed that the supplier significantly influenced the beta diversity of the samples at the genus level (*p*-value = 1e−3 for bacteria, archaea, and fungi). The different gut parts also showed differences in the microbiota between both suppliers, although the PERMANOVA test only showed significant differences for bacteria (*p*-value = 1e−3). As reported previously in Fig. 1, genera such as *Alistipes* and *Desulfovibrio* were more abundant in the hindgut, while others such as *Enterococcus* were less present in the hindgut. In P1 and P2, bacterial genera such as *Saccharimonadales*, *Enterococcus* and *Micrococcus* and fungal genera such as *Cutaneotrichosporon* and *Umbelopsis* were present in both suppliers. In addition, the archaeal genus *Methanobrevibacter* was detected in both suppliers along P2. Among P3 and P4, several overlapping bacterial genera were found, such as *Dysgonomonas*, *Tyzzerella* and *Alistipes*, and fungal genera such as *Oidiodendron*, *Penicillium* and *Saitozyma*. In addition, genera common to all four parts of Supplier S1 included *Saccharimonadales*, *Bacteroides* and *Gordonia* among the bacterial genera and *Candida*, *Apiotrichum* and *Humicola* among the fungal genera (Fig. 2a and Supplementary Dataset 3).

### Cellulose degradation

**Fig. 3:**
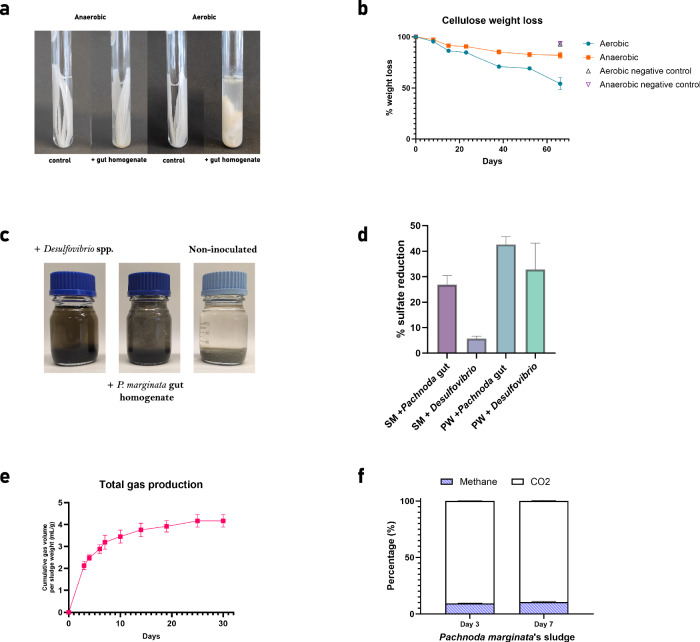
Potential biotechnological applications of *Pachnoda marginata* gut microbiota in cellulose degradation, sulfate bioremediation and methane production. *Cellulose biodegradation*: (**a**) Representative results of the assay incubated for 66 days at 25 °C under aerobic conditions (right) and anaerobic conditions (left). **b** Percentage of cellulose weight loss after the incubation period. Negative control samples were only measured at the end of the incubation period. The mean and the standard error of the mean (SEM) of three replicates per group are shown. *Sulfate reduction assay*: (**c**) Experimental set-up after the incubation time (40 days) in synthetic medium (SM). **d** Percentage of sulfate reduction in SM and sulfate polluted water (PW) at the end of the assay. The mean and the SEM of two replicates per group are shown. *Anaerobic digestion*: (**e**) Total gas production (mL/g initial sludge) in piston probers by *P. marginata* gut homogenate incubated at room temperature for 30 days in triplicate. The mean and the SEM of three replicates per group are shown. **f** Percentage of methane production compared to CO_2_ production (taking summatory of both = 100%) at day 3 and 7 of incubation in samples containing as sludge the *P. marginata* gut homogenate. The mean and the SEM of three replicates per group are shown.

**Fig. 4:**
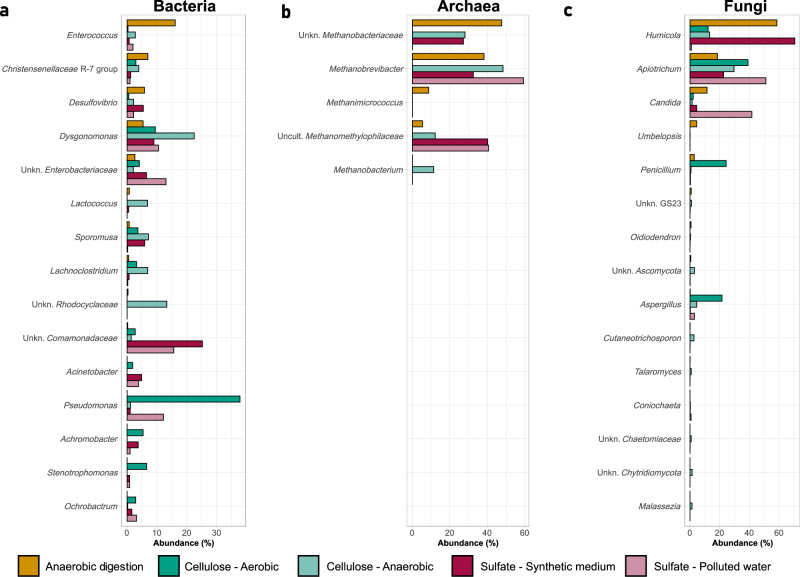
Community composition of bacteria, archaea, and fungi in the different assays at the end of the incubation period. Bacterial (**a**), archaeal (**b**) and fungal (**c**) communities detected at the end of the incubation period in each assay: anaerobic digestion (yellow); cellulose degradation in aerobic (dark green) and anaerobic (light green) conditions; sulfate reduction in synthetic sulfate-rich medium (dark red) and in sulfate-rich polluted water (light red). The 15 most abundant genera on average of all samples from the three different assays are shown here (in the case of archaea only 5 genera were detected in total).

In the cellulose degradation test, after two months of incubation of cellulose strips with *P. marginata* gut homogenate, aerobic and anaerobic cultures lost, on average, 45.7% and 17.9% of cellulose weight, respectively. Non-inoculated controls showed a 7.1% weight lost in aerobic and 6.5% in anaerobic conditions (Fig. 3a, b). Substantial variations in the microbial community exposed to cellulose at the end of the incubation period in both conditions were found (Fig. 4). Common aerobic genera such as *Pseudomonas*, *Stenotrophomonas* and *Achromobacter* were detected in high relative abundance under aerobic culture, but not under anaerobic conditions. Similarly, bacteria of the genus *Lactococcus* and the families *Rhodocyclaceae* and *Tannerellaceae* were abundant in the anaerobic culture and were not detected in the presence of oxygen (Supplementary Dataset 3). The archaeal genera identified in the enriched microbiota under anaerobic conditions were, in order of abundance, *Methanobrevibacter*, an unknown genus of the family *Methanobacteriaceae*, an uncultured genus of the family *Methanomethylophilaceae* and *Methanobacterium*. No archaeal sequences were detected under aerobic conditions. As for the fungi found, in both conditions *Apiotrichum* was the most abundant and a significant presence of *Humicola* was detected. The main differences found were the higher relative abundance of the genera *Penicillium* and *Aspergillus* under aerobic conditions and of *Cutaneotrichosporon* and *Malassezia* under anaerobic conditions.

### Sulfate bioremediation

The sulfate bioremediation assay also revealed positive results regarding the ability for sulfate reduction of the gut microbiota of *P. marginata*. Two different sulfate-rich solutions were inoculated with the gut homogenate of the larvae of this insect, a synthetic sulfate-rich media (SM) as well as a sulfate-rich polluted water from oil industry (PW). Inoculation with *Desulfobrivio* species, well known for its metabolic capability for sulfate reduction, was used as positive control. Both media turned black after 3 days of incubation due to iron sulfide production (Fig. 3c). The microbial community developed from the inoculum with *P. marginata* gut content in these sulfate-rich media was able to reduce sulfates in a 27% and 43% in SM and PW respectively, thus outperforming the positive control inoculated with *Desulfobrivio* (6% in SM and 33% in PW) (Fig. 3d). Regarding the microbiota composition developed after 40 days of incubation in this sulfate-rich solutions at room temperature, differences were found particularly in the fungal taxonomic profiles. The predominant bacterial genera, after the incubation period, in both the SM and the PW media were *Dysgonomonas* and two unidentified genera of the families *Comamonadaceae* and *Enterobacteriaceae*. In contrast, *Sporomusa* and *Desulfovibrio* were more abundant in the synthetic medium bioremediation assay, while *Pseudomonas* and *Ochrobactrum* were more abundant in the incubation with the industrial polluted water. The predominant archaea in both conditions were *Methanobrevibacter* and an uncultivated genus of the family *Methanomethylophilaceae*, while the main difference found among substrates was the detection of an uncultivated genus of the archaeal family *Methanobacteriaceae* only after the incubation with sulfate-rich synthetic medium. Regarding fungi, *Humicola* represented up to 70% of the relative abundance after the incubation with synthetic sulfate-rich medium, but barely reached 1% in the sulfate-rich polluted water. In the latter, the presence of *Apiotrichium* and *Candida* was higher (Fig. 4).

### Methanogenic activity of *P. marginata* gut content

To assess methanogenic activity, anaerobic digestion experiments were carried out in piston probers at room temperature as described in “Methods” using gut homogenates from *P. marginata* larvae. Total gas accumulation followed an exponential trend for the first week of incubation and slowed down as of day 7 (Fig. 3e). Anaerobic digestions with the *P. marginata* homogenate showed methane production, although in a low ratio compared with CO_2_ (<9%, Fig. 3f). In addition to methane and carbon dioxide, carbon monoxide and hydrogen were also measured but the contents were negligible.

Regarding the microbial composition, in the anaerobic digestion assay using *P. marginata* gut homogenate as sludge, at the end of the incubation period the most abundant bacterial genus was *Enterococcus* with almost more than 15% relative abundance. Other important genera were the R-7 group of the family *Christensenellaceae*, and the genera *Desulfovibrio* and *Dysgonomonas*, which exceeded 5% each. As for the presence of archaea, genera of the family *Methanobacteriaceae*, *Methanobrevibacter* and another unidentified one, represented more than 85% of the total number of archaea (Supplementary Dataset 3). The other two archaeal genera identified were *Methanimicrococcus* and an uncultured one from the family *Methanomethylophilaceae*. The most abundant fungal genus was *Humicola* with a relative abundance of almost 60% of total fungi. *Apiotrichium* and *Candida* also exceeded 10% each (Fig. 4).

When the microbial composition at the end of the incubation period of the three tests was compared, it was found that some of the genera described as prominent in the anaerobic digestion assay (such as bacterial genera like *Ruminococcus* and *Candidatus* Tammella and some genera belonging to the *Clostridia* class, together with archaeal genus *Methanimicrococcus*) were differentially more present in this assay than in the cellulose degradation and sulfate reduction assays (*p*-adjusted < 0.05) (Supplementary Fig. 2a). Similarly, two bacterial genera typically involved in cellulose degradation, *Cellulomonas* and *Lachnoclostridium*, were more abundant in the cellulose degradation test samples (Supplementary Fig. 2b), although not statistically significant (*p*-adjusted > 0.05). In the sulfate reduction assays, two genera belonging to the *Devosiaceae* and *Anaerovoracaceae* families were significantly more abundant compared to the other two assays (Supplementary Fig. 2c).

## Searching for genes involved in cellulose degradation, methanogenesis, and sulfate reduction

**Fig. 5:**
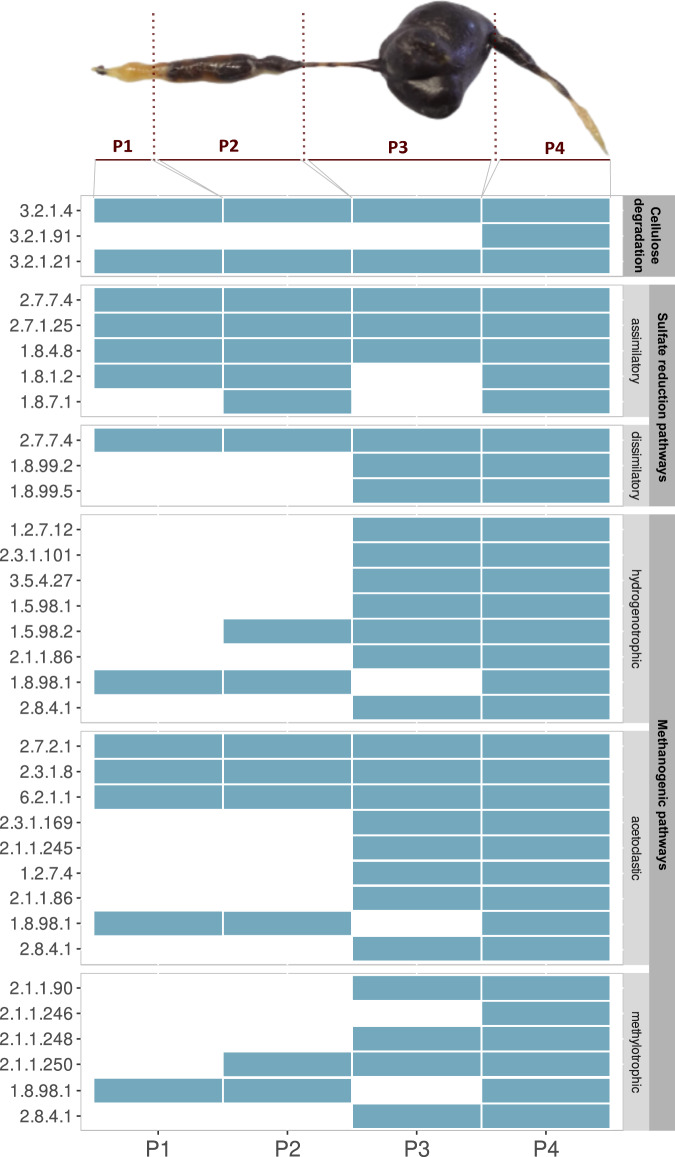
Presence-absence patterns (light blue—presence; white—absence) of genes of interest present in each assembly, grouped by metabolic activity: cellulose degradation, sulfate reduction pathways (assimilatory and dissimilatory) and methanogenesis (acetoclastic, hydrogenotrophic and methylotrophic pathways). EC numbers 1.12.98.2 (hydrogenotrophic methanogenesis), EC 2.1.1.249 and EC 2.1.1.247 (methylotrophic methanogenesis) did not retrieve any significant hit in any part of the gut with the filtering criteria used in the annotation with KofamScan. A complete list of all the EC numbers found in each gut section can be found in Supplementary Dataset 4.

**Fig. 6:**
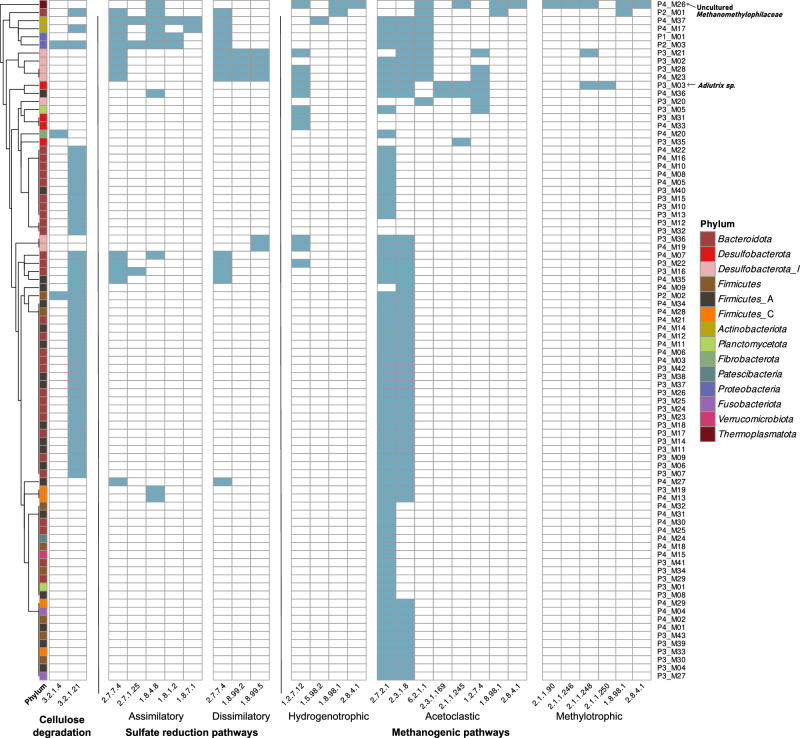
Presence-absence patterns (light blue—presence; white—absence) of genes of interest present in each MAG, grouped by metabolic activity: cellulose degradation, sulfate reduction (assimilatory and dissimilatory) and methanogenesis (acetoclastic, hydrogenotrophic and methylotrophic pathways). EC numbers 3.2.1.91 (cellulose degradation), 2.3.1.101, 3.5.4.27, 1.5.98.1, 1.12.98.2, 2.1.1.86 (hydrogenotrophic methanogenesis), 2.1.1.86 (acetoclastic methanogenesis), and 2.1.1.249, 2.1.1.247 (methylotrophic methanogenesis) were not found in any recovered MAG with the filtering criteria used in the annotation with KofamScan. The dendrogram to the left shows the hierarchical clustering of the MAGs based on their gene absence/presence profile.

Metagenomic analysis of the four sections of *P. marginata* gut was performed by filtering and assembly of the metagenomic reads and subsequent functional annotation of the obtained assemblies. Then, genes encoding enzymes involved in cellulose degradation, sulfate reduction and methanogenic pathways were searched in each of the four sections (Fig. 5) and all the pathways were found to be complete along the hindgut (P3 and P4). In the case of methylotrophic methanogenesis, due to the restriction parameters used for the annotation with KofamScan, the key activity EC 2.1.1.247, the methyltransferase that forms the substrate for EC 2.8.4.1 which catalyzes the last step in methanogenesis, was not retrieved from metagenomic data. However, when the annotation was revised manually by BLAST, sequences for EC 2.1.1.247 activity were found to be present in one of the recovered archaeal MAG P4_M26, suggesting then that the methylotrophic methanogenesis pathway was complete in the hindgut of the larvae. Furthermore, in methanogenic pathways, only generic enzymatic activities like heterodisulfide reductase EC 1.8.98.1, acetate kinases such as EC 2.7.2.1, EC 2.3.1.8 and EC 6.2.1.1 were present in the foregut, the aerobic section of the gut. Therefore, the hydrogenotrophic, acetoclastic and methylotrophic methanogenic pathways were found to be complete in the anaerobic hindgut section. Regarding sulfate reduction, the assimilatory pathway was present homogenously along the whole gut while genes of the dissimilatory sulfate reduction pathway (EC 1.8.99.2 and EC 1.8.99.5), related to anaerobic respiration by using sulfate as electron acceptor, were only present in the hindgut section. Lastly, genes for beta-glucosidase (EC 3.2.1.21) and cellulase (EC 3.2.1.4) were present homogeneously in all sections of the gut whereas the cellulose 1,4-beta-cellobiosidase (EC 3.2.1.91) was only found in the last part of the hindgut (Fig. 6).

In addition, these genes of interest were specifically targeted in the recovered high and good-quality metagenome-assembled genomes (MAGs) derived from the assemblies. In the hindgut, a much higher amount of high and good-quality MAGs were obtained, with up to 43 MAGs in P3 and 37 in P4, whereas in the foregut and midgut only 1 and 3 MAGs were obtained, respectively. These MAGs were taxonomically classified so that it was possible to determine which microbial taxon possessed the gene in question (Supplementary Dataset 5). Analogous to the search for these genes in the assemblies, their presence in each MAG was also checked. With respect to cellulose degradation, MAGs possessing the beta-glucosidase (EC 3.2.1.21) mainly belonged to the bacterial phyla *Bacteroidota* and *Firmicutes_A*. Cellulase (EC 3.2.1.4) was annotated in one MAG belonging to *Proteobacteria*, another to *Fibrobacterota* and lastly one classified as *Firmicutes*. With regard to sulfate reduction, the complete dissimilatory pathway was exclusively present in *Desulfobacterota_I* MAGs, which harbored the sulfate adenylyltransferase (EC 2.7.7.4), adenylylsulfate reductase (EC 1.8.99.2) and sulfite reductase dissimilatory type (EC 1.8.99.5). The latter enzyme is commonly used as a molecular marker to detect microorganisms capable of anaerobic respiration by using sulfate or sulfite as terminal electron acceptors^18^. As for the analysis of the presence of genes involved in anaerobic digestion, the most interesting MAG was P4_M26, belonging to the archaeal family *Methanomethylophilaceae*, which annotation was refined manually by BLAST to verify the presence of methanogenic pathways (Supplementary Dataset 6). P4_M26 held a methylotrophic pathway able to use methanol and methylamine to produce methane as well as the acetoclastic methanogenesis pathway. Related to this genus, as shown before, an uncultured *Methanomethylophilaceae* genus was present in the gut microbiota of both suppliers (Fig. 2) and was also found as one of the main archaea enriched at the end of the incubation period in the anaerobic digestion assay, anaerobic cellulose degradation assay and both sulfate-rich medium, PM and SM, in which the sulfate reduction ability was tested (Fig. 4). In addition, another MAG, P3_M03, which its closest genus identification is *Adiutrix*, presented some activities from the acetoclastic and methylotrophic methanogenesis. This suggested that it harbors the Wood-Ljungdahl pathway (WLP), for the anaerobic synthesis of acetyl-CoA from CO_2_. The whole repertoire of activities for the WLP (Table 1) were also inferred manually by BLAST in P3_M03 MAG (Supplementary Dataset 7). However, cellulose degrading enzymes were not identified in MAG P3_M03.

**Table 1:** EC numbers of enzymes involved in cellulose degradation, sulfate reduction and methanogenesis (hydrogenotrophic, acetoclastic and methylotrophic pathways)

Activity	EC numbers	Source
Cellulose degradation	3.2.1.4, 3.2.1.91, 3.2.1.21	Sikora et al.^62^
Sulfate reduction—assimilatory pathway	2.7.7.4, 2.7.1.25, 1.8.4.8, 1.8.1.2, 1.8.7.1	Zhou et al.^63^; Li et al.^64^
Sulfate reduction—dissimilatory pathway	2.7.7.4, 1.8.99.2, 1.8.99.5	Zhou et al.^63^; Li et al.^64^
Methanogenesis—hydrogenotrophic pathway	1.2.7.12, 2.3.1.101, 3.5.4.27, 1.5.98.1, 1.12.98.2, 1.5.98.2, 2.1.1.86, 2.8.4.1, 1.8.98.1	Sikora et al.^62^ ; Metacyc: superpathway of methanogenesis^65^
Methanogenesis—acetoclastic pathway	2.7.2.1, 2.3.1.8, 6.2.1.1, 2.3.1.169, 2.1.1.245, 1.2.7.4, 2.1.1.86, 2.8.4.1, 1.8.98.1	Sikora et al.^62^ ; Metacyc: superpathway of methanogenesis^65^
Methanogenesis—methylotrophic pathway	2.1.1.90, 2.1.1.246, 2.1.1.248, 2.1.1.249, 2.1.1.250, 2.1.1.247, 2.8.4.1, 1.8.98.1	Sikora et al.^62^ ; Metacyc: superpathway of methanogenesis^65^
Wood-Ljungdahl pathway	1.17.1.10, 6.3.4.3, 3.5.4.9, 1.5.1.5, 1.5.1.54, 2.1.1.258, 1.2.7.4, 2.3.1.169	MetaCyc: Reductive acetyl coenzyme A pathway I (homoacetogenic bacteria)^66^

## Discussion

In the present work, we report how the anatomically segmented gut of the scarab beetle larvae of *Pachnoda marginata* holds specific sequential microbial communities, which we have studied by amplicon sequencing of the 16S rRNA gene (archaeal and bacterial community) and ITS2 region (fungal population). Only a few reports have been published in the past in this regard, and they focused mainly on the study of the genus *Pachnoda* in general or the species *P. ephippiata* in particular^13,19^.

The high degree of differentiation among foregut, midgut and hindgut in terms of microbiota, has previously been described in *Pachnoda* genus but restricted only to the microbial shift among midgut and hindgut and the hindgut-specific microbiota^13,19^. In regard to the bacterial community, several of the most abundant genera found in *P. marginata* larval gut (i.e. *Bacteroides*, *Tannerella*, *Dysgonomonas*, *Alistipes*, *Ruminococcus*, *Clostridia* and *Sporomusa)* were also the main identified genera in *P. ephippiata* larvae by Andert et al. in 2010^13^.

We found clear trends in the variation of some bacterial abundances along the intestine of *P. marginata* larvae. The facultative-anaerobic genera *Bacillus*, *Enterococcus* and *Serratia* are more abundant in the foregut and midgut and disappear in the hindgut community. Thus, there is a total shift to a predominance of obligate-anaerobic, fermentative bacteria in the hindgut, where *Bacteroides*, *Alistipes, Desulfovibrio, Cand*. Soleaferrea*, Clostridia, Oscillospirales, Tyzzerella*, and the *Christensenellaceae* R-7 group dominate the bacterial community. This dramatic change in the microbial community is consistent with the described functions of the midgut and hindgut sections, the former being predominantly where enzymatic digestion takes place, and the latter behaving as a fermentation chamber^20^.

Ebert et al.^20^ proved, by analyzing the bacterial community of 21 coprophagic dung beetle species from the Scarabaeidae family, that the hindgut-microbial diversity was more dependent on host phylogeny and gut morphology than the diet or the environment these insects live in. As those authors suggested, hindgut morphology appears to be a key factor driving the microbial community. In their study, within the coprophagic dung beetles, larvae of the genus *Cephalodesmius* appeared to have a hindgut microbiota that more closely resembles other types of detritivores such as humus-feeding scarab larvae (i.e. *Pachnoda*) and termites, all of them sharing the characteristic of having the anterior hindgut dilated as a fermentation chamber^21^, rather than other coprophagic genera such as *Onthophagus*, which lacks the hindgut dilatation (Fig. 6 in Ebert et al.^20^). Accordingly, they described in three species of the dung beetle genus *Cephalodesmius* that the hindgut core microbiome shared, as the top five most abundant OTUs, the ones belonging to *Alistipes (Bacteroidetes), Cand*. Soleaferrea*, Tyzzerella* (*Bacillota* -formerly *Firmicutes*) and two *Desulfovibrio* sp. (*Pseudomonadota*; *Deltaproteobacteria*), which are all amongst the most abundant genera in all hindguts of the *P. marginata* samples analyzed in the present study. In contrast, the top five most abundant OTUs in the hindgut of other dung beetles of the genus *Onthophagus* (lacking the hindgut dilatation), do not match any of those described for *Cephalodesmius*^20^ and *P. marginata* from this study.

The main microbial key-players are present in the gut of *P. marginata* larvae regardless of the source from which they are obtained. When comparing larvae purchased from different suppliers, the predominant genera in all the three groups (bacteria, archaea and fungi) were shared among providers. However, we found remarkable differences, mainly in terms of archaeal and fungal diversity, when larvae from two different providers were compared, being significantly higher in larvae from Supplier 2. Differences among providers may be driven by growth conditions and feeding, although the influence of diet on the microbial community has been previously studied in *P. marginata* and *P. epiphiata* and was discarded as a key driver of the bacterial population^13^. These differences in total diversity can play an important role when the microbial communities from the gut of *P. marginata* are used for the purpose of strain isolation or as a source of enzymatic activities of interest for biotechnological and industrial purposes.

Under rearing conditions, *P. marginata* has a fiber-rich diet mainly consisting of coconut fiber and peat which is similar to their natural substrate also rich in cellulosic and lignocellulosic components. Degradation of these compounds is mainly attributed to the gut microbial community^22^. Therefore, we tested the cellulose degradation potential of a gut homogenate of *P. marginata* and our results showed that aerobic conditions outperformed the anaerobic conditions, being the degradation rate 45.7% and 17.9% respectively. Lemke et al.^22^ also described that the degradation of paper disks by inoculating the gut content of *P. ephippiata* only happened in aerobic conditions and not under anoxic or alkaline conditions. Members of the bacterial genera *Pseudomonas*, *Stenotrophomonas* and *Achromobacter* as well as the fungal genera *Penicillium* and *Aspergillus*, which are not present among the most abundant genera in the normal gut microbial community, are the ones significantly increasing their abundance in the cellulose degradation assay in aerobic conditions, suggesting they may play a role in this activity. Interestingly, in our metagenomic data, beta-glucosidase (EC 3.2.1.21) and cellulase (EC 3.2.1.4) genes were found to be homogeneously distributed throughout the gut regardless the oxygen availability (Fig. 5). Strains with hemi-cellulolytic activities have previously been isolated from *P. marginata* larval gut, such as the facultatively anaerobic bacterium *Xylanimonas pachnodae*^23,24^. Xylanase- and beta-1,4-endoglucanase-encoding genes have been described in this species^25,26^.

We have also demonstrated considerable potential for sulfate reduction by the *P. marginata* gut microbiota. Sulfate reduction rate by this insect species was first studied by Dröge et al. in 2005^17^ and proved 21-fold higher than the one from the termite *Mastotermes darwiniensis*, being termites previously known to harbor a rich community of sulfate-reducing bacteria (SRB), mainly dominated by *Desulfovibrio* species^27–29^. In the present study, at a fine scale of the intestinal compartments, we showed that, as expected, *Desulfovibrio* species are virtually absent in the more aerobic parts of the gut (foregut and beginning of midgut) and increase significantly their abundance in the anaerobic hindgut of *P. marginata* larvae, following the same exact pattern in both larvae from different suppliers. Furthermore, in the metagenomic analysis, the MAGs belonging to *Desulfobacterota* phylum are the ones carrying the complete dissimilatory sulfate reduction pathway and are also only present in the hindgut. *Desulfovibrio* species isolated from termite guts have shown the ability to either reduce sulfate or oxidize sulfide, which would allow the completion of sulfur cycle in the hindgut of the larval intestine^28^. Therefore, Kuhnigk et al.^28^ suggested that by running the complete sulfur cycle, *Desulfovibrio* species contribute to the oxidation of typical fermentation products (produced by other microorganisms in the community) to acetate which could then be used by the insect host as a carbon source. Probably due to the low availability of oxygen in the hindgut, oxidation of the acetate by other microorganisms would not play a prominent role and would thus remain available to the insect host. In addition, this cycle also allows sulfide reoxidation, hence decreasing the highly toxic H_2_S which could be harmful if accumulated in the termite gut^17^. Finally, also a role in nitrogen availability was described for *Desulfovibrio* species in termite guts due to their potential for nitrogen fixation^28^.

Regarding the archaeal community and methanogenic activity, *Methanobrevibacter* and an unknown genus of *Methanobacteriaceae* were the most abundant archaea in the larvae’s gut from both suppliers as well as the most abundant genera in the enriched community after the anaerobic digestion assay. *Methanobrevibacter* is commonly found in human gut microbiomes^30^ and has been shown as the most abundant methanogen when analyzing the archaeome across the animal kingdom^31^. Hence, we proved that archaeal diversity was highly influenced by the source of the larvae and besides the low diversity of methanogens observed for the larvae from Supplier 1; the larvae from Supplier 2 also contained *Methanosarcina, Methanobacterium, Methanothermobacter* and *Methanomassiliicoccus*, among others, which according to Thomas et al.^31^, are amongst the rarest methanogenic lineages, which can be found across the animal kingdom in the respective gut archaeomes. In the biogas industry, *Methanosarcina* is usually one of the most abundant archaea in bioreactors and it is considered a high-performance methanogen due to its metabolic versatility, since it is able to display all pathways of methanogenesis^32,33^. On top of that, the recovered MAG P4_M26, which its closest identity was an uncultured member of the archaeal family *Methanomethylophilaceae*, also found in larvae from both suppliers, carried out a complete set of genes for both the methylotrophic pathway and the acetoclastic pathway. This MAG was as well one of the most enriched taxa at the end of the sulfate reduction assay. Our results are in contrast with previous reports since the *Methanomethylophilaceae* family has been described before as an uncultured archaeal lineage in the *Methanomassiliicoccales* order of strictly H_2_-dependent methylotrophic methanogens^33^, which suggests that MAG P4_M26 may belong to a new uncultured archaeal family.

Finally, we recovered MAG P3_M03, which its closest genus identity is *Adiutrix*, and presents the genetic set for the WLP for reductive acetogenesis from CO_2_ and H_2_. This genus has never been cultivated since it has been described as an endosymbiont of termite gut flagellates^34^. In accordance with this finding, it is interesting to highlight that some of the homologous proteins to the WLP in P3_M03 have been also inferred in a deltaproteobacteria endosymbiont of the gutless oligochaete worm *Olavius algarvensis* and its suggested role is also the autotrophic CO_2_ fixation^35^. Furthermore, *Desulfovibrio* species have also been described as protist endosymbionts in termite guts^36^. Therefore, this finding in *P. marginata* opens the door towards the study of the role that eukaryote endosymbionts may play in the gut microbiota of this beetle larvae, which, to the best of our knowledge, has not been studied before.

Taken together, our results show that *P. marginata* gut has an anatomically, physiologically and microbiologically hyperdifferentiated gut in which a range of parameters, particularly oxygen availability, shape microbial communities that are central for the insect metabolism. Such diverse communities and their activities, from an anthropocentric perspective, hold potential for a range of industrial applications, including sulfate reduction and, to a lesser extent, cellulolytic and methane production activities.

## Methods

### Insects

Third-instar larvae of *Pachnoda marginata* (subsp. *peregrina*) were purchased from a commercial supplier (HarkitoReptile, Madrid, Spain = Supplier 1 in this study) and maintained at 25 °C and 60% humidity in a diet composed of coconut fiber and peat (purchased from the same supplier) on a 1:1 proportion. Larvae were kept on these conditions a maximum of 4 days before sampling the gut. Larvae from this supplier were used for all the experiments carried out in this study. For the comparison of the gut microbiota depending on the provider, third-instar larvae were also purchased from “La Ferme Aux Coleos” (Cherbourg-en-Cotentin, France = Supplier 2 in this study) and maintained in the same conditions as described above.

### Gut dissection and DNA extraction

*P. marginata* larvae were dissected by using sterile dissection tools and the complete digestive tract was obtained independently from each individual. Firstly, one digestive tract was divided into 12 parts: F1 and F2 (Foregut); M1-M4 (Midgut); H1.1-H2.3 and H3-54 (Hindgut) to perform preliminary studies of the microbial composition along the gut (Fig. 1a) (Engel and Moran, 2013)^21^.

Due to the similarity in microbial composition of the parts constituting the foregut, midgut and hindgut individually, the final gut samples of the study were divided into 4 parts to simplify the processing of the data: P1 (F1 and F2, Foregut); P2 (M1-M4, Midgut); P3 (H1.1-H2.3, first half of Hindgut) and P4 (32-H5, Last half of Hindgut). Therefore, 3 larvae from Supplier 1 (HarkitoReptile, Madrid, Spain) and 3 other larvae from Supplier 2 (La Ferme Aux Coleos, Cherbourg-en-Cotentin, France) were dissected. Individual gut sections were separately introduced into sterile microcentrifuge tubes and 100 µL of sterile saline solution (composition in g/L: 9 g NaCl) were added to each tube and the content was thoroughly ground using a pestle until a homogeneous solution was obtained. Then, a volume of 100 µL of the resulting homogenized samples were processed for DNA extraction by using the DNeasy PowerSoil Pro Kit (QIAGEN GmbH, Ref. 47014) following the manufacturer’s protocol.

### Amplicon sequencing and taxonomic analysis

The DNA extraction performed of each type of sample in this study is described in their specific section in “Methods”. All the DNA extractions were validated by DNA quantification (Qubit 2.0 Fluorometer, Qubit 1X dsDNA HS Assay kit, Thermo Fisher, USA). Three different amplicon sequencing runs were performed in order to obtain the bacterial, archaeal and fungal taxonomic profiles. For the study of the bacterial communities of the 12 parts of the gut, the conserved regions V3 and V4 (459 bp) of the 16S rRNA gene in each of the 12 parts were then amplified using forward and reverse primers: 5′-TCG TCG GCA GCG TCA GAT GTG TAT AAG AGA CAG CCT ACG GGN GGC WGC AG 3′ and 5′- GTC TCG TGG GCT CGG AGA TGT GTA TAA GAG ACA GGA CTA CHV GGG TAT CTA ATC C-3′^37^; for the study of bacterial and archaeal communities in the suppliers comparison analysis, the cellulose degradation test, the anaerobic digestion assay and the sulfate reduction assay, the amplification of the V4 region was performed using primers 515F (5′-GTGCCAGCMGCCGCGGTAA-3′) and 806R (5′-GGACTACHVGGGTWTCTAAT-3′)^37^ which allowed to the identification of both, bacterial and archaeal species by the same sequencing run. DNA amplicon libraries were generated using a PCR cycle consisting of an initial denaturation step at 95 °C for 5 min; the annealing step consisting of 30 cycles (95 °C for 30 s, 54 °C for 30 s, 72 °C for 30 s); and the extension at 72 °C for 10 min. For the study of all fungal communities, the ITS2 region of fungal nuclear ribosomal DNA (rDNA) was amplified using universal primers ITS3_KYO2 (5’-GATGAAGAACGYAGYRAA-3′) and ITS4_KYO1 (5′-TCCTCCGCTTWTTGWTWTGC-3′)^38^. In this case, the amplification of this region was performed by an initial denaturation step at 95 °C for 32 min; the annealing step consisted of 28 cycles (95 °C for 30 s, 58 °C for 30 s, 72 °C for 30 s); and the extension at 72 °C for 5 min. To confirm the amplification of the 16S rRNA gene and ITS2 fragment amplicons, 1% agarose gel electrophoresis was used to monitor the PCR products. Subsequently, dsDNA was purified from the PCR products and resuspended in 10 μL MilliQ water. For 16S rRNA gene Sanger sequencing, the samples were tagged using the BigDye Terminator v3.1 Cycle Sequencing Kit (Applied Biosystems, Carlsbad, CA, USA), while the ITS2 rDNA amplicon was labeled with Illumina sequencing adapters and dual index barcodes (Nextera XT index kit v2, FC-131-2001). Prior to sequencing, the libraries were pooled and normalized. Then, the pool of indexed amplicons was loaded onto the MiSeq reagent cartridge v3 (MS-102-3003), and supplemented with 10% PhiX control to optimize sequencing quality. Finally, the Illumina MiSeq sequencing system was used to perform paired-end sequencing (2 × 300 bp) at the Sequencing Service (SCSIE) of the University of Valencia.

QIIME 2 software (v2021.2.0)^39^ was used to analyze the raw Illumina sequences. Read quality was assessed using the Demux plugin, and sequences were then trimmed and joined, corrected, and clustered into amplicon sequence variants (>99.9% similarity) using the Dada2 pipeline built into Qiime2. The classify-sklearn plug-in of QIIME2 was used to assign the taxonomy of each sequence variant, with the SILVA database (v138)^40^ as the reference for 16S rRNA gene assignment and the UNITE database (v8.2)^41^ as the reference for the fungal ITS2 rDNA region.

Various R packages and functions were used for data analysis. phyloseq package (v1.30.0)^42^ and hill taxa function from the hillR package (v0.5.2)^43^ were used to generate alpha diversity plots based on Hill numbers *q* = 0 (species richness), *q* = 1 (exponential of Shannon’s entropy), and *q* = 2 (inverse Simpson index). The results of each supplier’s richness were presented as mean ± standard error of the mean (SEM). These alpha diversity analyses were performed with the ASV counts rarefied to the smallest sample size using the rarefy_even_depth function from the phyloseq package. PCoA plots were generated using the plot_ordination function, also from phyloseq, with the Bray-Curtis dissimilarity metric as the distance method. All heat maps were constructed using the amp_heatmap function from the ampvis2 library (v2.7.2)^44^. For the rest of the plots, the R package ggplot2 (v3.4.0)^45^ was used. For statistical analyses, the PERMANOVA test was used to check the existence of significant differences (*p*-value < 0.05) between the microbial composition of the two suppliers in terms of beta diversity, using the adonis2 function from the vegan R package (v2.6.4)^46^. The MaAsLin2 R package (v1.0.0)^47^ was used to perform the differential abundance analyses between taxa, with the following parameters: min_abundance = 0, min_prevalence = 0.05, max_significance = 0.05, normalization = ‘None’, transform = ‘LOG’, analysis_method = “LM”, correction = “BH”, standardize = FALSE.

### Cellulose degradation test

Glass test tubes were filled with 10 strips of 0.5 × 5 cm of Whatman filter paper (Cat. 11392805) previously weighed. The assay tubes with the paper strips were autoclaved and afterwards 7 mL of sterile M9 medium (Composition for 1 L of final volume: 200 mL M9 Salts Solution 5X (Serva Electrophoresis GmbH, Ref. 48505), 2 mL MgSO_4_ 1 M and 0.1 mL CaCl_2_ 1 M) were added to each tube. Then, 3 larvae of *P. marginata* from Supplier 1 were dissected in sterile conditions (following the procedure explained above) to obtain the complete digestive tract. Separately, each digestive tract was resuspended in 4 mL of PBS and grinded with a pestle until a homogeneous inoculum was obtained. For the inoculation of the assay tubes, 50 µL of each ground sample was added to the corresponding tube. Twelve identical tubes for each *Pachnoda* gut were prepared as well as six control tubes without inoculum. Half of the tubes were incubated in anaerobic conditions using the BD GasPack™ EZ System (Ref. 260683) and the rest were incubated in aerobic conditions. All the tubes were incubated at 25 °C and no agitation during a period of 66 days. Replicates were analyzed at 6 different time points, the first three were measured weekly and the last three every two weeks. To analyze the microbial composition developed in each condition, a gentle agitation by vortexing at low speed was carried out and 1 mL of the supernatant in each tube was collected for DNA extraction. The aliquots were centrifuged at 12,000 rpm for 10 min in order to obtain the microbial pellet which was then processed by using the DNeasy PowerSoil Pro Kit (QIAGEN GmbH, Ref. 47014) following the provider’s instructions to extract the DNA. The DNA was finally sent for amplicon sequencing, as described in the section above. Cellulose degradation was quantified by filtering the remaining content of the tubes on pre-weighed filter paper, left to dry overnight at 60 °C and finally weighed to obtain the weight of the remaining cellulose.

### Sulfate bioremediation assay

A synthetic sulfate rich media (SM) (Postgate medium, DSM medium 63; https://www.dsmz.de/microorganisms/medium/pdf/DSMZ_Medium63.pdf) as well as sulfate-rich polluted water (PW) from oil industry (containing 7500 ppm/L sulfate and adjusted to pH 7.2 by adding NaOH 4 M) used in a previous study^48^, were used as source of sulfate. The assay was conducted in duplicate in both sulfate-rich solutions using as inoculum the gut homogenate of *P. marginata* and a positive control inoculated with a sulfate-reducing bacterial (SRB) consortium of *Desulfovibrio* species previously described in a sulfate bioremediation study^48^, which was requested from the authors. The two specimens of *P. marginata* from Supplier 1 were dissected as previously explained and an inoculum of 100 µl from each were added to two 0.1 L borosilicate glass bottles filled with 100 mL of SM as well as to two bottles containing 100 mL of PW. Each bottle contained 35 g of sterile glass beads (0.4 cm diameter) as solid matrix. The same procedure was followed to inoculate the positive controls by adding 100 L of the SRB consortium culture at OD_600_ = 0.5. A non-inoculated bottle of each sulfate-rich medium were also included as negative controls. All the bottles were incubated at room temperature for 40 days without agitation and leaving the cap slightly open to allow gas pressure to escape. After the incubation, the content of each bottle was homogenized by agitation and 50 mL were transferred to new plastic bottles that were sent to Laboratorios Tecnológicos del Levante S.L. (Paterna, Spain) for sulfate quantification. The remaining 50 mL from each bottle were filtered by a vacuum filter and the DNA extraction from the complete filter was carried out by using the DNeasy PowerSoil Pro Kit (QIAGEN GmbH, Ref. 47014) and following the provider’s instructions. The DNA was sent for amplicon sequencing as explained in the “Amplicon sequencing and taxonomic analysis” section.

### Anaerobic digestion assay

To analyze methanogenic activity from the gut content of *P. marginata*, glass piston probers of 100 mL volume were used (Poulten & Graf GmbH, Wertheim, Germany). *P. marginata* larvae from Supplier 1 were dissected to extract the complete gut until enough volume for the assay was obtained (approximately 30 guts per replicate were needed). The assay was carried out in triplicate. All the guts for each replicate were ground together under sterile conditions in a tube by the use of a pestle until a homogeneous sample was reached. Then, for each replicate, 34 mL of intestinal homogenate were filled into a piston prober. Pistons were greased to ensure gas tightness (KWS-Schliff-Fett, Carl Roth GmbH, Karlsruhe, Germany). The total incubation period was 30 days at room temperature and the total gas produced was monitored in accumulated volume (mL) during this period. When enough gas was accumulated in each sample, gas was pumped into headspace vials via a cannula for further GC analysis to determine the CH_4_ and CO_2_. Sampling times for the gas to perform GC analysis were day 3 and 7 of incubation. The cannula was connected to the outlet valve of the piston prober via a short hose connection (1 cm in length). Before the sampling, the cannula and the hose connection were shortly flushed with gas from the piston probers. The headspace vials were prefilled with an acidic displacement liquid and were provided by Eurofins Umwelt Ost GmbH (Germany). The composition of the displacement liquid was prepared according to the German Institute for Normalization (DIN 38414-8): 30 mL of sulfuric acid, H_2_SO_4_ (p = 1.84 g/mL), are added to 1 L of distilled water; 200 g of sodium sulfate decahydrate, Na_2_SO_4_·10H_2_O, are dissolved in this mixture with gentle heating. The solution was colored red-orange by adding a few drops of methyl orange solution (0.1 g of methyl orange sodium salt dissolved in 100 ml of distilled water). The displacement liquid should be stored at room temperature. At low temperatures, sodium sulfate can crystallize out, which must first be redissolved by heating the mixture. After displacement of liquid in the headspace vials, the gas filled vials were taken to Eurofins for further analysis. The concentration (vol.-%) of methane, carbon monoxide, carbon dioxide and hydrogen using a mobile gas chromatograph (“Mobiler Gaschromatograph MobilGC”, ECH Elektrochemie Halle GmbH, Germany) was analyzed. The device was a 2-channel gas chromatograph (GC) equipped with a thermal conductivity detector (WLD) and a flame ionization detector (FID). The measurement was performed in accordance with the German guideline DIN 51872-5: 1996-08 (Deutsches Institut für Normung e.V). Furthermore, 100 µL of the remaining biomass from each reaction were used for DNA extraction by using the “Power Soil Pro DNA extraction kit” following the manufacturer’s protocol and sent for amplicon sequencing as described previously.

### Shotgun metagenomics analysis

A *P. marginata* larvae gut was dissected in four parts (P1, P2, P3 and P4) and the DNA was extracted as described before in the “Gut dissection and DNA extraction” section. The extracted DNA underwent a series of steps to prepare it for whole genome sequencing. First, the resulting DNA fragments polished and had A-tails were added. Next, adaptors (sequences 5′-AGA TCG GAA GAG CGT CGT GTA GGG AAA GAG TGT AGA TCT CGG TGG TCG CCG TAT CAT T-3′ and 5′-GAT CGG AAG AGC ACA CGT CTG AAC TCC AGT CAC GGA TGA CTA TCT CGT ATG CCG TCT TCT GCT TG-3′) were ligated to the fragments. The fragments were then amplified by PCR, and the resulting products were purified using the AMPure XP system for library preparation. The size distribution of the libraries was assessed using an Agilent 2100 Bioanalyzer, and the libraries were quantified using real-time PCR. Finally, sequencing was performed on the Illumina NovaSeq 6000 platform (2×150 bp). Adapter sequences were then trimmed using Cutadapt (v1.15)^49^, and filtered for quality and potential contaminants using BBDuk, a tool included in BBTools (v38.50; parameters: qtrim=lr trimq=20 maq=20 minlen=75) (Joint Genome Institute; https://sourceforge.net/projects/bbmap/). Human-derived reads (GRCh38.p13 index) were further filtered using the --un-conc option of the bowtie2 tool (v2.3.4.1)^50^. After filtering the raw reads, a quality control check was performed using FastQC (v0.11.5) (http://www.bioinformatics.babraham.ac.uk/projects/fastqc) on the filtered metagenomic sequences, which were then assembled using MEGAHIT (v1.2.9)^51^. QUAST (v5.0.2)^52^ was used to assess the quality of genome assemblies. MetaBAT (v. 2.12.1)^53^ and MaxBin (v. 2.2.7)^54^ were then used for binning with default parameters. DAS Tool (v. 1.1.3)^55^ was used to select a non-redundant set of the MAGs. The quality of the selected MAGs was then assessed using CheckM (v1.1.3)^56^, and they were classified into three categories: HQ (high quality; completeness ≥ 90% and contamination ≤ 5%), GQ (good quality; completeness ≥ 80% and contamination ≤ 10%) and LQ (low quality; completeness ≤ 80% or contamination ≥ 10%). The LQ MAGs were discarded from the analysis. Taxonomic annotation of the HQ and GQ MAGs was performed using the Genome Taxonomy Database Toolkit (GTDB-Tk, v2.1.1)^57^ with GTDB R07-RS207 as reference data.

### Search for genes of interest

Functional annotation was performed on both the metagenomic assemblies and the HQ and GQ MAGs obtained for each gut section, in order to search for genes involved in cellulose degradation, anaerobic digestion and sulfate reduction. For this purpose, the KEGG orthology and hidden Markov model (HMM)-based tool KofamScan (v1.3.0)^58^ was used, and only annotated genes with an e-value ≤ 1e−5 and a score higher than the predefined threshold were retained. In addition, hits were filtered to include only those related to the activities of interest (Table 1). Clustered heat maps showing presence or absence of the activities were generated using the R package pheatmap (v1.0.12) with the default parameters^59^. For MAGs of interest, certain activities were manually revised by conducting reciprocal BLAST (blastp; v2.9.0+)^60^ searches against protein sequences linked to these activities in the BRENDA database^61^, substantial outcomes were obtained by setting specific filtering parameters (coverage > 80, bitscore > 40, e-value ≤ 1e−6) and output data can be found in Supplementary Datasets 6 and 7.

### Limitations

While the present study provides in-depth insights into the microbial community inhabiting the gut of *P. marginata* larvae and their potential in industrial applications, several limitations should be acknowledged. Foremost among these limitations is the small sample size utilized in the study of the bacterial population in *P. marginata* gut divided in 12 parts (results shown in Fig. 1) in which only an individual gut was analyzed. This first analysis should be seen as a case study, and as the starting point that guided us to determine the potential and diversity of the gut of *P. marginata*, as well as it allowed us to decide in how many parts to divide the digestive tract in following replicates from different suppliers. Furthermore, we consider that for a preliminary analysis, the division of the gut into 12 parts is very detailed and the results are consistent among all the 12 sections, as demonstrated by the PERMANOVA test. In addition, it should be also noted that the oxygen levels along the larval gut were not measured for this study with *P. marginata*. However, the low oxygen availability in the gut of coleopterans, and particularly in *Pachnoda* species, has been measured previously as shown by Lemke et al.^22^ study with larvae of the closely related species *P. ephippiata*. Also our results regarding the microbial profiles and metabolic functions (e.g. methanogenesis) of the different gut sections are in concordance with this well-known differences in oxygen content along the insect gut.

There are also certain aspects that may need to be studied in more detail on the functional assays for cellulose degradation, anaerobic digestion, and sulfate reduction. In these assays, complete guts were used to inoculate the different replicates and only the final adapted microbial community was studied, while the different gut sections were not tested separately. We consider that we partially overcame this deficiency by including the metagenomic analysis of the intestine divided into four parts, where we have searched for the enzymatic pathways of these activities of interest in each gut section. In addition, neither culturomics nor functional analyses of individual microbial species were carried out as they were beyond the scope of the present work.

Consequently, future studies with expanded sample sizes and detailed studies on the roles played by individual microbial species are encouraged to further validate and extend our findings. Despite these limitations, our study represents an important step towards understanding the complex microbial communities inhabiting the highly compartmentalized gut of *P. marginata*, laying the groundwork for future research in this field.

### Supplementary information

Supplementary_figures

Supplementary Dataset 1

Supplementary Dataset 2

Supplementary Dataset 3

Supplementary Dataset 4

Supplementary Dataset 5

Supplementary Dataset 6

Supplementary Dataset 7

## Data Availability

The datasets generated for this study can be found in online repositories. Raw reads are available at NCBI’s Sequence Read Archive (SRA) (Bioproject Accession PRJNA1023051).

## Code availability

All the code used in this study for data analysis has been uploaded to GitHub: https://github.com/danieltorsil/Pachnoda-marginata-gut-microbiome
