# Supplementary material for: The highly differentiated gut of Pachnoda marginata hosts sequential microbiomes: microbial ecology and potential applications
Source: NPJ Biofilms Microbiomes. 2024 Jul 31;10:65. doi: 10.1038/s41522-024-00531-7 (PMC11291753; doi:10.1038/s41522-024-00531-7)
Supplement: Supplementary file 1 — Supplementary_figures [file 41522_2024_531_MOESM1_ESM.pdf]

Supplementary material

“The highly differentiated gut of *Pachnoda marginata* hosts sequential microbiomes: microbial ecology and potential applications.”

Àngela Vidal-Verdú, Daniel Torrent, Alba Iglesias, Adriel Latorre-Pérez, Christian Abendroth, Paola Corbín-Agustí, Juli Peretó*, Manuel Porcar*

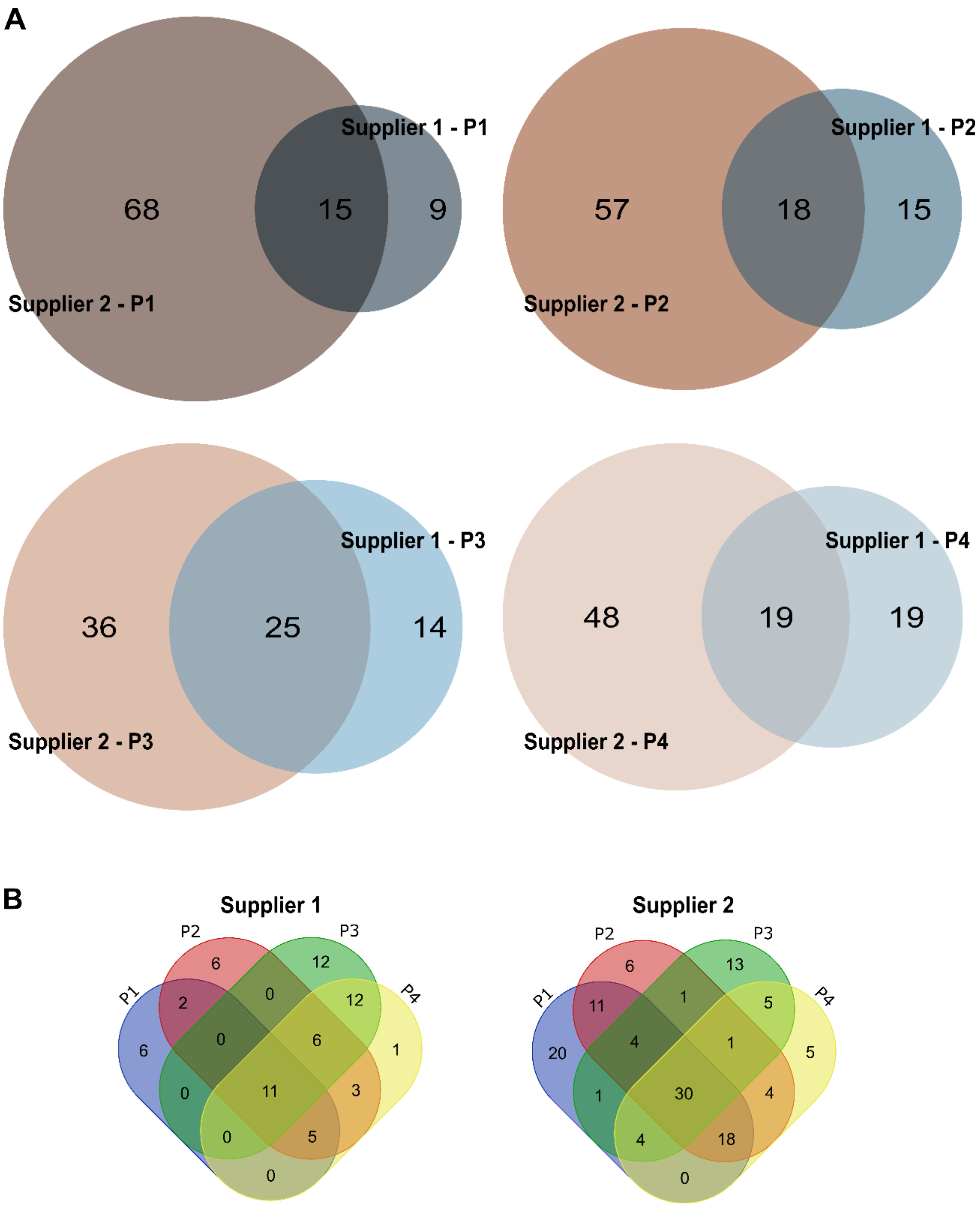

Supplementary figure 1. Venn diagrams showing the defined genera (bacterial, fungal, and archaeal) present exclusively in each supplier and those shared by both for each part of the gut **(A)**; Microbial taxa shared by each part of the gut, divided by supplier **(B)**.

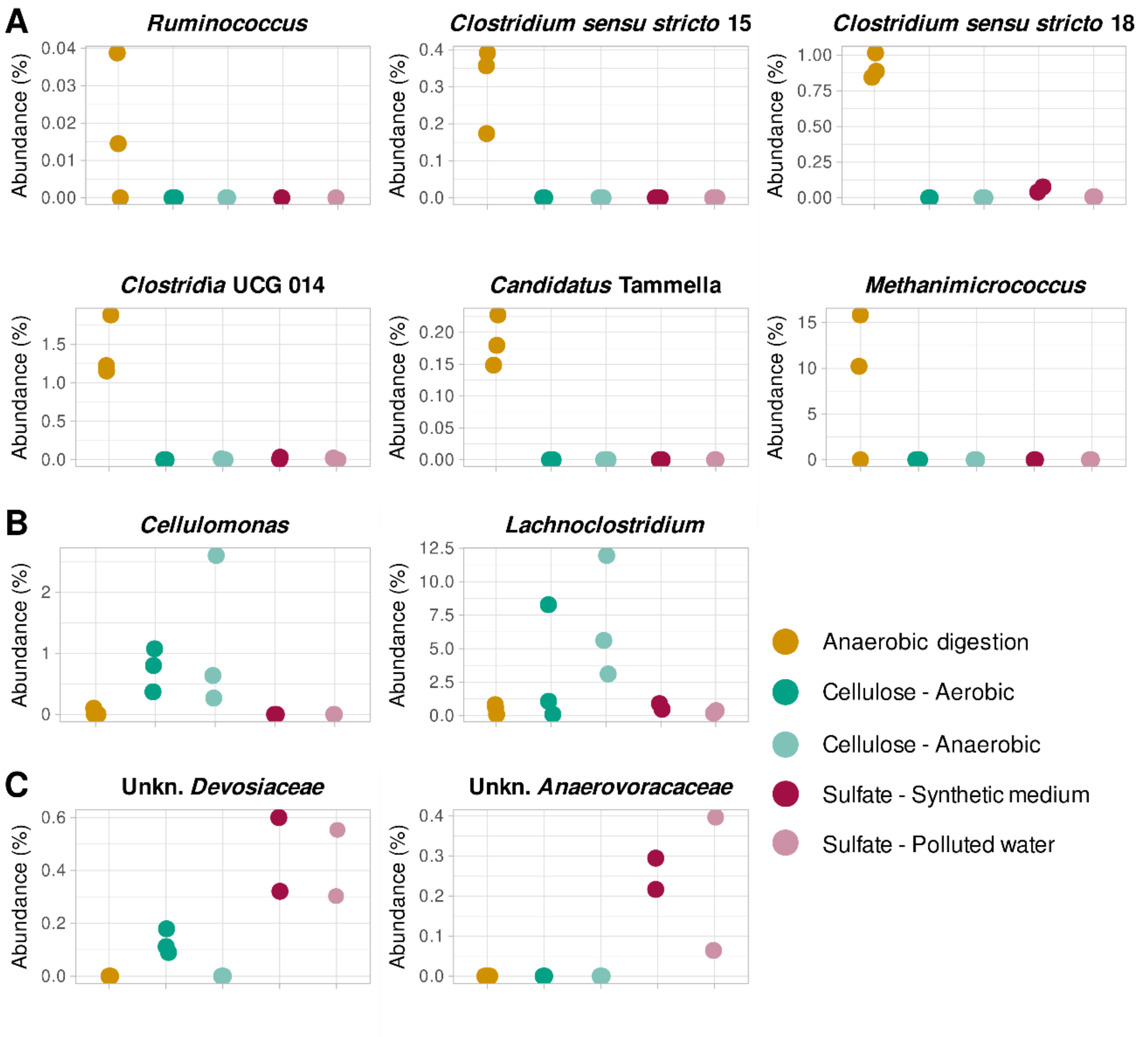

Supplementary figure 2. Relative abundances (%) of some genera that were significantly more abundant in each of the three assays (**A:** anaerobic digestion; **B:** cellulose degradation; **C:** sulfate reduction), at the end of the incubation period.
